# Repositioning baloxavir marboxil as VISTA agonist that ameliorates experimental asthma

**DOI:** 10.1007/s10565-024-09852-x

**Published:** 2024-02-10

**Authors:** Jian-wen Di, Yi-xin Wang, Rui-xue Ma, Zhi-jie Luo, Wen-ting Chen, Wan-mei Liu, Ding-yi Yuan, Yu-ying Zhang, Yin-hao Wu, Cai-ping Chen, Jun Liu

**Affiliations:** 1 New Drug Screening Center, China Pharmaceutical University, Nanjing, 210009 China; 2 State Key Laboratory of Natural Medicines, China Pharmaceutical University, Nanjing, 210009 China; 3 Chongqing Innovation Institute of China Pharmaceutical University, Chongqing, 401135 China

**Keywords:** VISTA, Asthma, Macrophage polarization, Baloxavir marboxil

## Abstract

V-type immunoglobulin domain–containing suppressor of T-cell activation (VISTA), a novel negative checkpoint regulator, plays an essential role in allergic pulmonary inflammation in mice. Treatment with a VISTA agonistic antibody could significantly improve asthma symptoms. Thus, for allergic asthma treatment, VISTA targeting may be a compelling approach. In this study, we examined the functional mechanism of VISTA in allergic pulmonary inflammation and screened the FDA-approved drugs for VISTA agonists. By using mass cytometry (CyTOF), we found that VISTA deficiency primarily increased lung macrophage infiltration in the OVA-induced asthma model, accompanied by an increased proportion of M1 macrophages (CD11b^+^F4/80^+^CD86^+^) and a decreased proportion of M2 macrophages (CD11b^+^F4/80^+^CD206^+^). Further in vitro studies showed that VISTA deficiency promoted M1 polarization and inhibited M2 polarization of bone marrow–derived macrophages (BMDMs). Importantly, we discovered baloxavir marboxil (BXM) as a VISTA agonist by virtual screening of FDA-approved drugs. The surface plasmon resonance (SPR) assays revealed that BXM (KD = 1.07 µM) as well as its active form, baloxavir acid (BXA) (KD = 0.21 µM), could directly bind to VISTA with high affinity. Notably, treatment with BXM significantly ameliorated asthma symptoms, including less lung inflammation, mucus secretion, and the generation of Th2 cytokines (IL-5, IL-13, and IL-4), which were dramatically attenuated by anti-VISTA monoclonal antibody treatment. BXM administration also reduced the pulmonary infiltration of M1 macrophages and raised M2 macrophages. Collectively, our study indicates that VISTA regulates pulmonary inflammation in allergic asthma by regulating macrophage polarization and baloxavir marboxil, and an old drug might be a new treatment for allergic asthma through targeting VISTA.

**Supplementary Information:**

The online version contains supplementary material available at 10.1007/s10565-024-09852-x.

## Introduction

Asthma is a frequent long-term respiratory condition that impacts over 350 million individuals globally (Cottini et al. 2022). Over the past decades, the incidence and prevalence of asthma have increased, especially in children. Numerous drugs are available to treat asthma presently, including β2-adrenoceptor agonists, corticosteroids, and monoclonal antibodies targeting key cytokines (i.e., IL-5, IL-13, and IL-4) (Pedersen et al. 2011; Koski and Grzegorczyk 2020). These drugs significantly improved lung function, decreased exacerbation rates, and provide better asthma control. However, some patients still fail to obtain remission after medication (Kardas et al. 2022). Therefore, developing novel targets for asthma is crucial.

V-type immunoglobulin domain–containing suppressor of T-cell activation (VISTA) is a negative checkpoint regulator that belongs to the B7 family (Yuan et al. 2021). In addition to activated T cells, VISTA is also present on naïve T cells, which is different from CTLA-4 and PD-1 found on activated T cells only. Therefore, it is critical for keeping T-cell quiescence and peripheral tolerance (ElTanbouly et al. 2020). VISTA shows high expression levels in diverse groups of myeloid cells, such as monocytes, macrophages, and dendritic cells, with low expression in eosinophils and not present in B cells, mast cells, or basophils (Gao et al. 2017; Xu et al. 2019; Zhang and Xiao 2022; Ohno et al. 2018). Studies using VISTA-deficient mice or blocking monoclonal antibodies (mAbs) have demonstrated that VISTA is involved in tumor immune escape and autoimmune diseases (Ceeraz et al. 2017; Han et al. 2019; Wang et al. 2014). Notably, research findings suggest that VISTA serves as a ligand or a receptor (Wang et al. 2014; Flies et al. 1950), but the VISTA binding partners remain elusive. VSIG3 (V-set and immunoglobulin domain containing 3) and PSGL1 (P-selectin glycoprotein ligand 1) interact with VISTA, but their in vivo functional binding remains to be verified (Wang et al. 2019; Xie et al. 2021; Johnston et al. 2019).

In asthma, the Chen group and the Azuma group demonstrated that VISTA negatively regulates Th2-mediated experimental allergic asthma by either genetic ablation or antibody blockade of VISTA in mice (Ohno et al. 2018; Liu et al. 2018). The two groups mainly focused on the alteration of T-cell subpopulations during the allergen challenge. The Chen group found that pulmonary Tregs (CD4^+^Foxp3^+^) were significantly reduced in VISTA KO mice, while the Azuma group revealed CD4^+^ T cells in draining lymph nodes generating more IL-13 upon anti-VISTA mAb (MIH63) treatment (Ohno et al. 2018; Liu et al. 2018). Whether other types of immune cells are involved in mediating VISTA regulation of pulmonary inflammation in asthma remains to be elucidated. In addition, a VISTA agonistic antibody (4C11) could suppress allergic pulmonary inflammation in mice, indicating that targeting VISTA might be an attractive therapeutic strategy for allergic asthma (Liu et al. 2018). However, to our knowledge, no VISTA agonists have yet entered clinical trials. Thus, it would be of great significance to discover VISTA agonists from the FDA-approved drugs.

In this study, by using mass cytometry (CyTOF), we discovered that VISTA deficiency mainly affects the proportion of lung macrophages. Further studies showed that VISTA regulates macrophage polarization, as VISTA deletion significantly dramatically increases M1 polarization and inhibits M2 polarization both in vitro and in ovalbumin (OVA)-induced asthmatic mice model. Furthermore, we initially identified baloxavir marboxil (BXM), an antiviral therapeutic drug for influenza viruses (Yang 2019), as a VISTA agonist. Treatment with BXM significantly improved asthma symptoms in the OVA-induced asthma model, and this effect was significantly blocked by anti-VISTA antibody treatment. Collectively, these data indicate that VISTA activation ameliorates lung inflammation in the experimental asthma model through the regulation of macrophage polarizing, and baloxavir marboxil might be a promising treatment for allergic asthma through targeting VISTA.

## Materials and methods

### Reagents and antibody

BXM and BXA were from Jiangsu Weikaier MediTech Company, Ltd. (Nanjing, China). DEX was from Sigma-Aldrich (St. Louis, MO, USA). Anti-mouse VISTA antibody was bought from BioXell (13F3, New Hampshire, USA).

### Animals

Specific pathogen-free female VISTA knockout (KO) mice (BALB/c background) and wild-type (WT) mice at the age of 6–8 weeks were from the Shanghai Model Organisms Center (Shanghai, China). Mice were fed normally, with unrestricted access to food and water free of pathogens. All animal experiments were conducted at China Pharmaceutical University and were approved by the Animal Care and Use Committee of China Pharmaceutical University.

### Mouse model of experimental asthma

Mice were sensitized to 20 μg OVA (Sigma-Aldrich, USA) with 4 mg aluminum hydroxide (Thermo Fisher Scientific, Waltham, USA) gel by intraperitoneal injection (ip) on days 0 and 5. From day 12 to day 18, mice were challenged with 20 mL of 1% aerosolized OVA for 25 min. The BXM treatment was performed by daily oral administration at the dose of 50 mg/kg 1 h before the OVA challenge (days 12–18). The DEX treatment was performed by daily ip injection at the dose of 1 mg/kg 1 h before OVA challenge (days 12–18). The mAb treatment was performed by ip injection of 250 μg on days 12, 14, 16, and 18, 1 h before the OVA challenge.

### Mass cytometry (CyTOF) analysis of immune cells

Lung tissues pre-perfused with PBS were digested into single cells with a mixture of DNase I and collagenase I (37 ℃ for 30 min) and filtered through a 70-μm filter. Erythrocytes were lysed prior to the enrichment of immune cells using Percoll. Cells were blocked and stained with a mixture of metal-labeled antibodies targeting surface antigens at room temperature for 30 min. After washing and fixation for 20 min at room temperature (RT), a membrane break buffer was used to wash the cells before incubation of intracellular metal-labeled antibodies (30 min at RT). After washing for three times, the cells were subjected to the CyTOF system, and then, cell clustering analysis and dimensionality reduction analysis were performed. Metal-conjugated antibodies are shown in Supplementary Table S1.

### Lung histopathology

After being dehydrated, the right lung was fixed with 4% paraformaldehyde and embedded in paraffin. The lung sections (5 µm) were subjected to hematoxylin and eosin (H&E) staining or periodic acid–Schiff (PAS) staining. The following criteria were used to assess the degree of inflammation on a scale from 0 to 4: 0, none; 1, slight; 2, moderate; 3, marked; and 4, very marked (Fisher et al. 2005). The abundance of PAS-positive cells was scored as follows: 0, 0–10%; 1, 11–25%; 2, 26–50%; 3, 51–75%; and 4, 76–100% (Adams and Cydulka 2003).

### Assays of bronchoalveolar lavage fluid and sera

After being collected, bronchoalveolar lavage fluid (BALF) was promptly centrifuged (1200 rpm, 7 min). Levels of mouse IL-4, IL-5, IL-13, and IFN-γ in BALF were analyzed using ELISA kits (Biolegend, USA). The overall immune cells in BALF were examined under a microscope. Upon staining with Diff-Quick (Nanjing Jiancheng Bioengineering Institute, China), 200 cells were counted on each slide under a high-magnification microscope. Mice sera samples were collected, and IgE and OVA-specific IgE were detected by specific ELISA kits.

### Immunohistochemistry (IHC)

The lung tissue slices underwent high-pressure boiling in a citric acid buffer solution after being deparaffinized and rehydrated. After quenching endogenous peroxidase activity, the section was blocked and incubated with primary antibodies, including F4/80 (1:500 dilution, GB115301, Servicebio, Wuhan, China), CD86 (1:500 dilution, ab269587, Abcam, Cambridge, UK, USA), and CD206 (1:500 dilution, GB113497, Servicebio, Wuhan, China) overnight at 4 °C. Next, a secondary antibody was gently dropped onto the slides. Diaminobenzidine was added for the visualization of the antigens, followed by hematoxylin staining.

### Flow cytometry

Following a FACS buffer soak, single cells from mouse lungs were incubated with antibodies CD11b-PE-Cy7 (101216, BD Biosciences, USA), F4/80-Brilliant Violet 421 (123137, Biolegend, USA), CD86-FITC (105005, Biolegend, USA), Ly6C-APC (128015, Biolegend, USA), and CD206-PE (141705, Biolegend, USA). Stained cells were analyzed by CytoFLEX cytometer (Beckman, USA).

### Quantitative real-time PCR (qPCR)

Total RNA was extracted from lung tissues using TRIzol reagent and reversed (500 ng) using a commercial kit (HiScript II Q RT SuperMix for qPCR, Vazyme, Nanjing, China) according to the manufacturer’s instructions. 1 × of ChamQTM SYBR qPCR Master Mix (High ROX Premixed) (Vazyme, Nanjing, China) was used for the reaction according to the manufacturer’s instructions. The Supplementary Table S2 displays the primers. Genes were quantified using the comparative 2^−ΔΔCT^ method.

### Culture and polarization of bone marrow–derived macrophages (BMDMs)

Mouse BMDMs were obtained and grown in accordance with earlier studies (Liu et al. 2016; Li et al. 2020). Briefly, BMDMs were collected from 6-week-old female mice and maintained in DMEM replenished with 10% FBS and 20 ng/mL macrophage colony-stimulating factor (M-CSF, Cat#315–02-10, PeproTech, USA) for 7 days at 37 °C and under 5% CO_2_. The cell purity was first confirmed by flow cytometry analysis of F4/80 staining. Next, LPS (100 ng/mL) and IFN-γ (20 ng/mL) were used to induce M1 macrophage polarization, and IL-4 (20 ng/mL) and IL-13 (20 ng/mL) were used to induce M2 macrophage polarization.

### Visualization of docked ligands with VISTA

Procedure for virtual screening was established as we previously reported (Hu et al. 2021; Yang et al. 2023). Briefly, 3D structure homology modeling was established by submitting the extracellular domain of human VISTA (162 amino acids, UniProt: Q9H7M9) to the COACH online server. For drug repositioning, FDA-approved molecules (DRUGBANK, 2021 Dec) were applied for molecular docking. PyMOL (PyMOL, version 1.7.4.5, RRID: SCR_000305) was used to show the interactions between VISTA and molecules. The distance between amino acid residues and ligands ≤ 1 Å was marked as potential interactions.

### Surface plasmon resonance (SPR) measurements

The SPR test was carried out as we documented before (Yang et al. 2023; Li et al. 2022). Briefly, human VISTA protein diluted to 20 μg/mL with sodium acetate solution (pH = 5.0) was immobilized onto the CM5-type chip by amino coupling. The response values of compounds flowing across the CM5 chip surface were measured at 25 °C using a Biacore T200 (GE Healthcare, USA) instrument.

### Data and statistical analysis

Data were presented as mean ± SD with the number of observations shown in figure legends. The statistical significance between groups was analyzed by unpaired student’s *t*-test, with *P* < 0.05 being considered significant.

## Results

### OVA-induced allergic airway inflammation is exacerbated by VISTA deletion

**Fig. 1:**
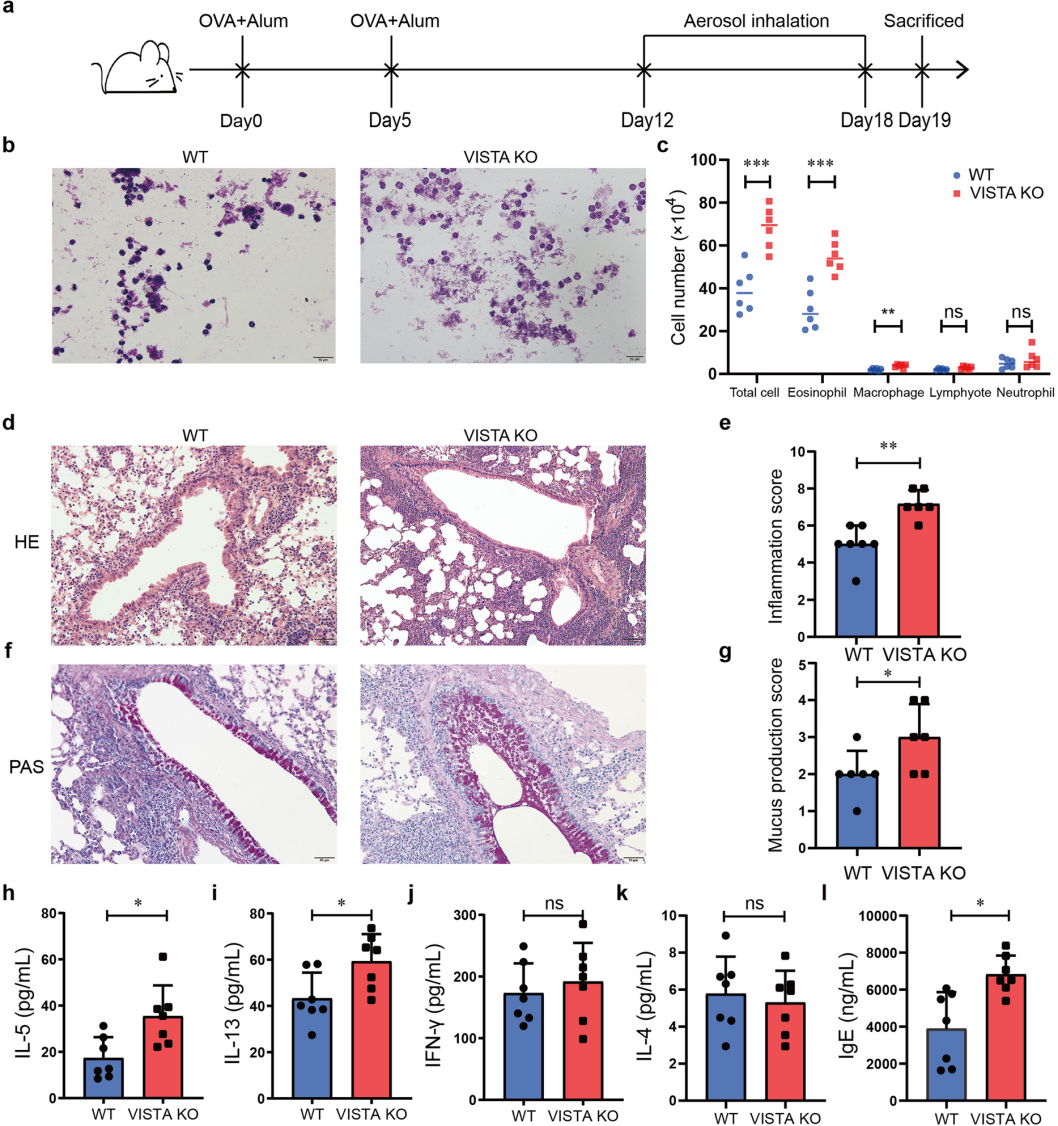
VISTA KO mice develop more severe asthma symptoms. **a** Experimental protocol to induce experimental asthma. **b**,** c** BALF was collected and stained for leukocyte counts by Diff-quick staining. **d**,** e** The lung images with H&E staining and lung inflammatory score from mice treated with OVA. **f, g** The lung images with PAS staining and mucus production score are shown. **h**–**k** The cytokine levels of IL-5 (**h**), IL-13 (**i**), IFN-γ (**j**), and IL-4 (**k**) in BALF were examined by ELISA. **l** Total IgE in serum was measured by ELISA. Data are shown as means ± SD (*n* = 6–7). All scale bars are 50 μm. ns: not significant, *P < 0.05, **P < 0.01, ***P < 0.001

First, we challenged mice with OVA to induce an experimental asthma model (Fig. 1a, Supplementary Fig. S1). The overall number of immune cells in the BALF was more elevated in the VISTA KO mice than in the WT mice, with eosinophils and macrophages significantly increased (Fig. 1b, c). Moreover, around the bronchiole, the VISTA KO mice showed increased inflammatory cells and PAS-positive goblet cells that produce mucin (Fig. 1d–g). Cellular cytokines known as T helper 2 (Th2) are essential to the pathogenesis of allergic asthma (Halim et al. 2012). We observed that the VISTA KO mice challenged with OVA revealed that BALF produced greater levels of IL-5 and IL-13 than WT mice (Fig. 1h, i), whereas the production of IL-4 and IFN-γ was not significantly altered (Fig. 1j, k), which is in line with other reports (Ohno et al. 2018; Liu et al. 2018). An increase in IgE serum level in the OVA-treated VISTA KO mice was also observed (Fig. 1l). Taken together, these data demonstrate that VISTA deficiency exacerbates OVA-induced asthma in mice.

### CyTOF shows that VISTA deficiency increased lung macrophage infiltration in OVA-induced asthma model

**Fig. 2:**
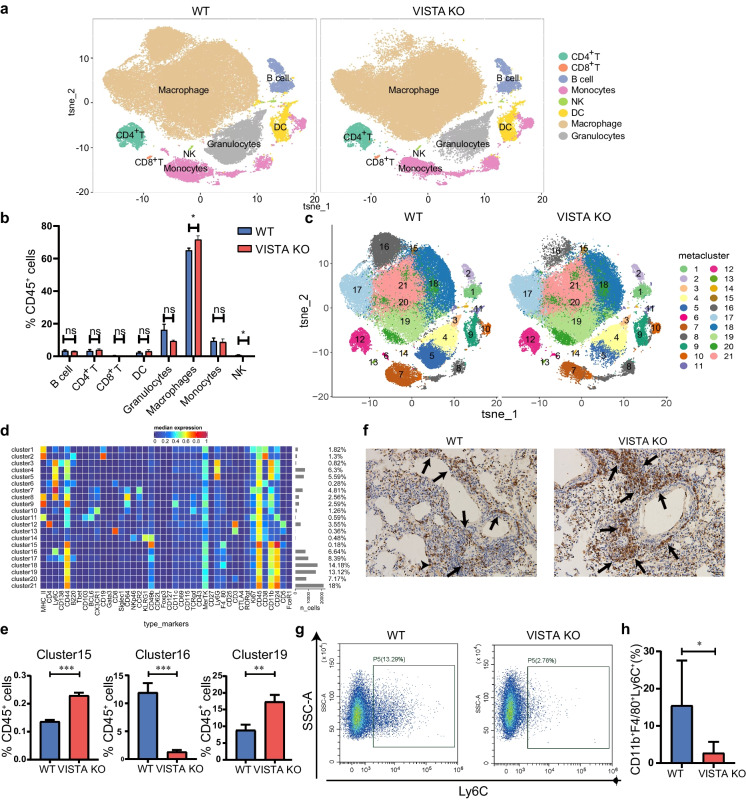
Mass cytometry analysis of lung immune cells in OVA-induced asthma model. **a** Representative t-SNE plots showing major immune cell subsets in lung tissue from WT and VISTA KO in OVA-induced asthma model. **b** Quantification of mass cytometry data showing abundance of lung immune cells from WT and VISTA KO mice. **c** Representative t-SNE plots of 21 cell clusters from WT and VITA KO mice: B cell, two clusters (1, 2); granulocytes, three clusters (3, 4, 5); monocytes, three clusters (6, 7, 8); DC, T cells, one cluster (9); NK cells, one cluster (14); macrophage, eight clusters (11, 15, 16, 17, 18, 19, 20, 21) (*n* = 3). **d** Heatmap of the expression of each protein in the 21 cell clusters. **e** Quantification of the macrophage cluster 15, 16, and 19 from WT and VISTA KO mice in OVA-induced asthma model (*n* = 3). **f** Representative images of IHC for F4/80 from WT and VISTA KO mice in OVA-induced asthma model (*n* = 4). Scale bars are 50 μm. **g** Representative flow cytometry plots of Ly6C^+^ macrophages. **h** Quantification analyses of Ly6C.^+^ macrophages (*n* = 6). ns: not significant, *P < 0.05, **P < 0.01, ***P < 0.001

To further investigate the cell types that are affected by VISTA deletion in the OVA-induced asthma model, we performed CyTOF analysis. A total of 8 clusters, CD4^+^ T cells (CD3^+^CD4^+^CD11b^−^), CD8^+^ T cells (CD3^+^CD8^+^CD11b^−^), B cells (CD19^+^B220^+^CD11b^−^), natural killer cells (NK, NKp46^+^CD49b^+^KLRG1^+^), macrophages (CD11b^+^F4/80^+^), dendritic cells (DCs, CD11b^+^CD11c^+^), monocytes (CD11b^+^Ly6C^+^Ly6G^−^), and granulocytes (CD11b^+^Ly6G^+^), were identified by the expression level of surface markers in different subgroups (Fig. 2a).

Our data showed that the total macrophages and NK cells differed significantly between the two groups (Fig. 2b). The overall count of macrophages experienced a notable rise (WT, 64.95% vs. KO, 71.59%), while NK cells (WT, 0.67% vs. KO, 0.29%) were significantly decreased in the VISTA KO group. Because NK cells, with a confusing role in asthma as reported, (Haspeslagh et al. 2018), make up a very small proportion of the CD45^+^ cells in the lung，and what is more, VISTA has low expression on NK cells (ElTanbouly et al. 2020), we speculate that NK cells may not the vital cell type that mediating VISTA's function. On the contrary, macrophage, with a high expression of VISTA, is the most abundant immune cell subset during the development of asthma and has a significant impact on allergic asthma (Deng et al. 2023; Miki et al. 2021; Song et al. 2018). Therefore, we further studied the macrophage subpopulations.

The macrophages were further subdivided into seven clusters (clusters 15–21), and the whole immune cells were consequently classified into 21 different clusters (Fig. 2c and d). We found that two subsets of macrophages, clusters 15 and 19, were significantly increased in the VISTA KO group (Fig. 2e). Interestingly, a subset of Ly6C^+^ macrophage (cluster 16) was reduced in VISTA KO mice (Fig. 2e).

Immunohistochemical studies also demonstrated a significant increase in F4/80^+^ macrophages in asthma lungs after VISTA deletion (Fig. 2f), which is in line with results identified by CyTOF. In addition, flow cytometry analysis also revealed a reduction in the CD11b^+^F4/80^+^Ly6C^+^ macrophage subset in VISTA KO mice throughout the OVA challenge (Fig. 2g, h), further convincing that our CyTOF results are credible. Taken together, our findings indicate that VISTA deficiency primarily increases macrophage infiltration in the murine model of allergic asthma.

### VISTA deficiency regulates macrophage polarization in OVA-induced asthma model

**Fig. 3:**
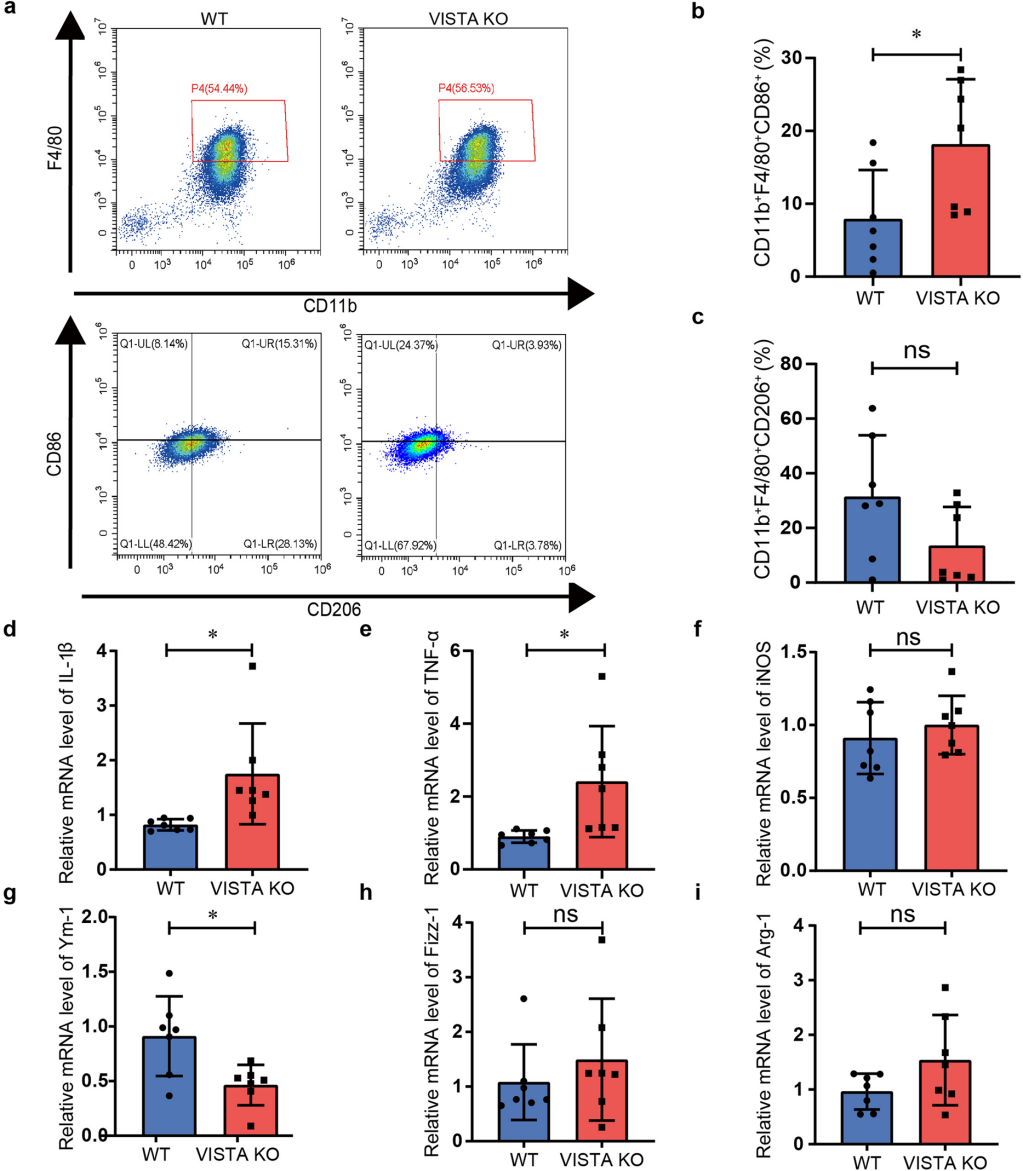
VISTA modulates macrophage phenotype in mouse asthma model. **a** Representative flow cytometry plots of M1 macrophages (CD11b^+^F4/80^+^CD86^+^) and M2 macrophages (CD11b^+^F4/80^+^CD206.^+^). **b**, **c** Quantification analyses of M1 and M2 macrophages. **d**, **i** The mRNA levels of M1 marker (IL-1β, TNF-α, iNOS) and M2 marker (Ym-1, Fizz-1, Arg-1) in lung tissues were determined by qPCR. Data are shown as means ± SD (*n* = 7). ns: not significant, *P < 0.05

Allergy asthma is significantly impacted by macrophage polarization (Saradna et al. 2018; Kuo et al. 2021). Upon recruitment, macrophages exhibit two distinct phenotypes: classically activated (M1) or alternatively activated (M2). M1 macrophages yield high expression of CD86, CD80, MHCII, TLR4, and iNOS and secrete elevated concentrations of inflammatory cytokines (e.g., IL-6, IL-12, IL-1β, and TNF-α) and chemokines (e.g., CCL2, CCL5), to recruit and activate T and B lymphocytes and to protect against bacteria and viruses (Saradna et al. 2018). M2 macrophages release cytokines that reduce inflammation (e.g., IL-10 and TGF-β) and down-regulate the immune response to control immune regulation and tissue remodeling (Rigamonti et al. 2014). Therefore, we wondered whether VISTA deficiency affects macrophage polarization. M1 and M2 macrophages were identified via an examination of flow cytometry. The gating strategies for the detection of the M1 to M2 macrophage ratio in asthma lungs are shown in Fig. 3a. We found that M1 macrophages (CD11b^+^F4/80^+^CD86^+^) were much higher in VISTA KO mice compared to the WT mice (Fig. 3b). In addition, M2 macrophages (CD11b^+^F4/80^+^CD206^+^) were decreased in OVA-induced VISTA KO mice (Fig. 3c). However, this was not statistically significant because of a large individual variation. Then, to further confirm M1/M2 phenotype polarization, we studied the M1-associated genes (TNF-α, IL-1β, iNOS) and M2-associated genes (Arg-1, Fizz-1, Ym-1) using qPCR. The findings demonstrated that in contrast to WT mice, the levels of M1-related genes IL-1β and TNF-α in asthmatic lungs of VISTA KO mice were significantly increased, and the levels of M2 macrophage phenotype gene Ym-1 were significantly decreased (Fig. 3d, e, g). However, the mRNA levels of the M1 marker iNOS and the M2 markers Fizz-1 and Arg-1 showed no discernible differences between the two groups (Fig. 3f, h, i). To sum up, these results suggest that VISTA deficiency affects macrophage polarization in the OVA-induced mouse asthma model.

### VISTA regulates macrophage polarization in vitro

**Fig. 4:**
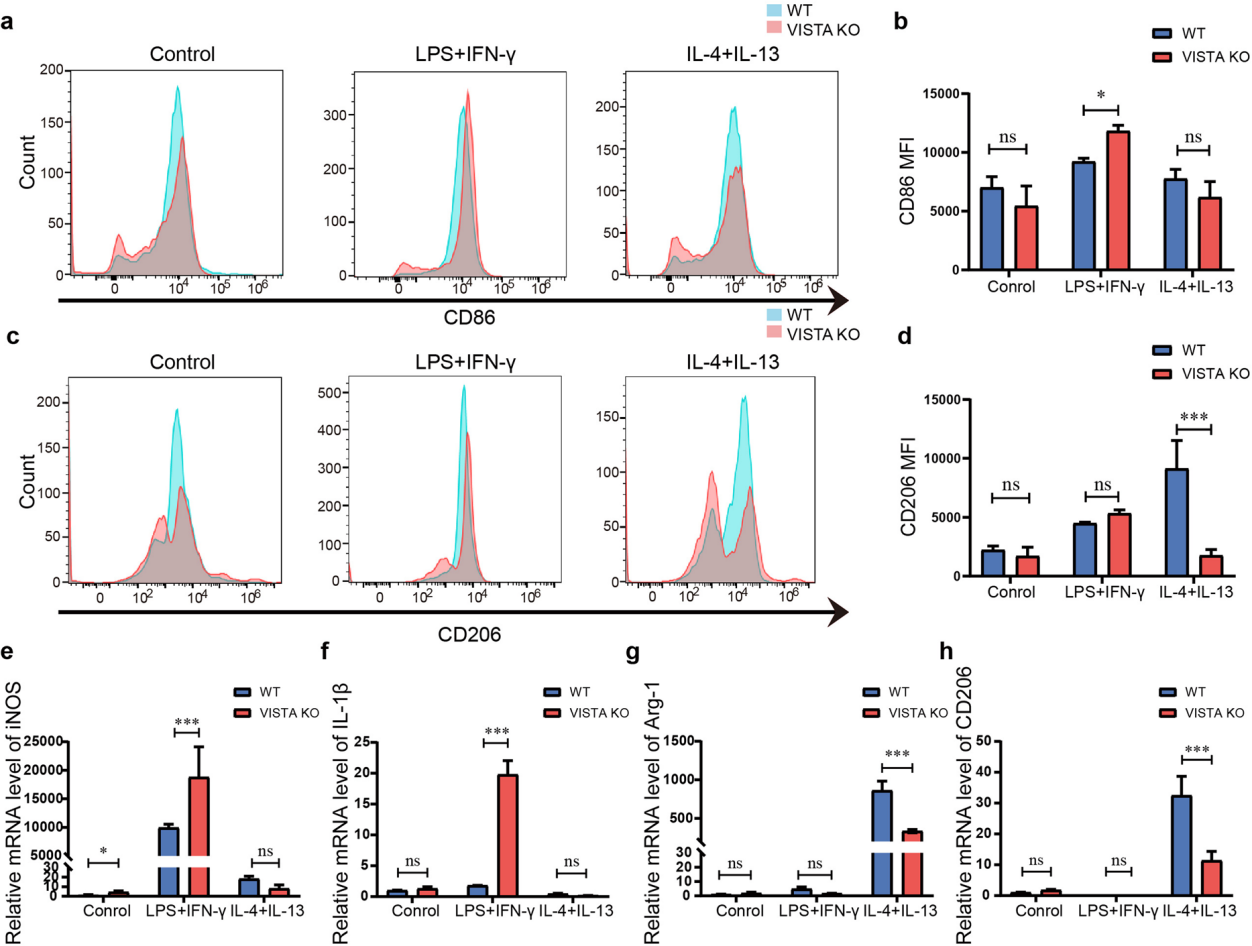
VISTA deficiency promoted M1 macrophage polarization and inhibited M2 macrophage polarization in vitro. **a**–**d** The expression of CD86 and CD206 in WT and VISTA KO BMDMs cultured in control medium (M0), LPS + INF-γ (M1), or IL-4 + IL-13 (M2) were determined by flow cytometry (**a** CD86 and **c** CD206) and quantified (**b** CD86 and **d** CD206). **e**–**h** The mRNA levels of iNOS (**e**), IL-1β (**f**), CD206 (**g**), and Arg-1 (**h**) were determined by qPCR in isolated BMDMs. ns: not significant. Data are shown as means ± SD (*n* = 3). *P < 0.05, **P < 0.01, ***P < 0.001

To determine whether VISTA deletion affects the macrophage polarization in the OVA-induced asthma model was primary or secondary, we studied macrophage polarization in BMDMs isolated from WT and VISTA KO mice. Flow cytometry assays revealed that loss of VISTA induced upregulation of CD86 expression during M1 polarization (LPS + IFN-γ) and downregulated CD206 expression during M2 polarization (IL-4 + IL-13) (Fig. 4a–d). Consistently, there was a considerable rise in M1 genetic markers, such as iNOS and IL-1β, and M2 genetic markers, such as Arg-1 and CD206, were reduced after VISTA deletion (Fig. 4e–h). This result agrees with a previous study showing that a VISTA agonistic antibody reprograms macrophages by negatively regulating macrophage upon pro-inflammatory stimuli (ElTanbouly et al. 2020). Thus, these results demonstrated that VISTA directly gets involved in the regulation of M1/M2 polarization.

### Discovery and characterization of VISTA agonist

**Fig. 5:**
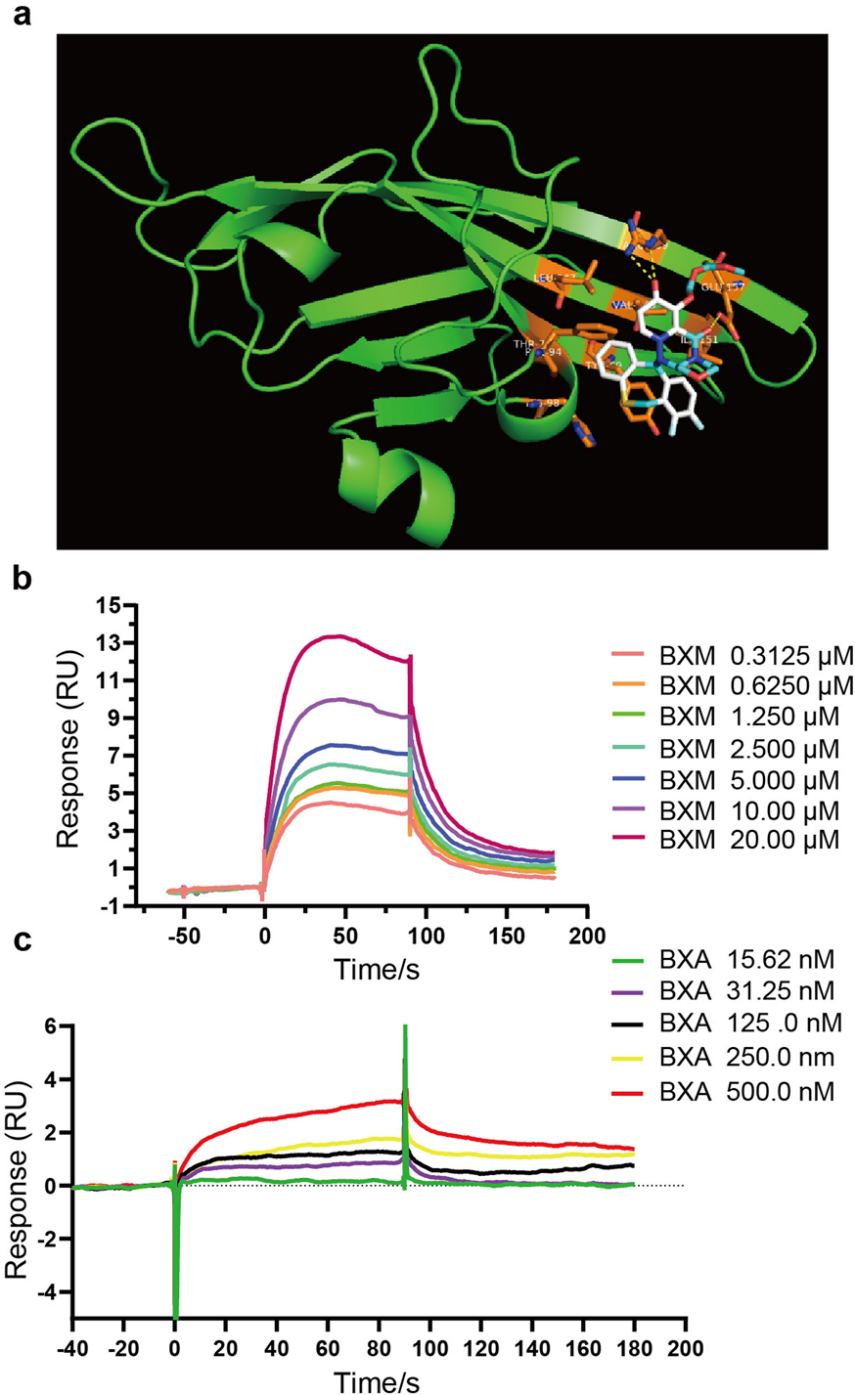
BXM binding with protein human VISTA. **a** Binding model of BXM and human VISTA-ECD protein (green). Predicted residues on the VISTA protein interacting with BXM are highlighted in orange. Hydrogen bonds between the ligand and residues are highlighted with a yellow dashed line. **b** Binding affinity was detected between human VISTA protein and BXM by SPR (*n* = 3). **c** Binding affinity was detected between human VISTA-ECD protein and BXA by SPR (*n* = 3)

As VISTA has a significant impact on the suppression of the experimental asthma model, then we explored whether VISTA small-molecule agonists could ameliorate airway inflammation. We built a homology model of the three-dimensional structure of VISTA and docked the VISTA virtual structure model with 2513 FDA-approved small-molecule drugs (DRUGBANK, 2021 Dec). After the virtual screening, we found that baloxavir marboxil (BXM) might be the candidate. The predicted residues of their interaction are shown in Fig. 5a and Table 1. Subsequently, we detected the *K*_*D*_ value of BXM/VISTA using the SPR method and identified BXM as a strong ligand (*K*_*D*_ = 1.07 µM, Fig. 5b). BXM is a prodrug for the treatment of influenza virus infection, and it will be metabolized into its active form in vivo, baloxavir acid (BXA) (Ando et al. 2021). Thus, we investigated whether BXA could bind to VISTA. The results showed BXA with a better binding affinity (*K*_*D*_ = 0.21 µM) than BXM (Fig. 5c). These results indicate that both BXM and its active metabolite BXA could directly bind to VISTA.

**Table 1:** The docked parameters of BXM interaction with human VISTA-ECD protein

Macromolecule	Hit ligands	Hydrogen bonds	Potential hydrophobic interactions	Predicted binding energy (kcal/mol)
Human VISTA	BXM	GLU-157, ARG-159	VAL-149, ILE-151, LEY-147, PHE-94, HIS-98, THR-71, LEU-147, TYR-69	− 7.3

**Fig. 6:**
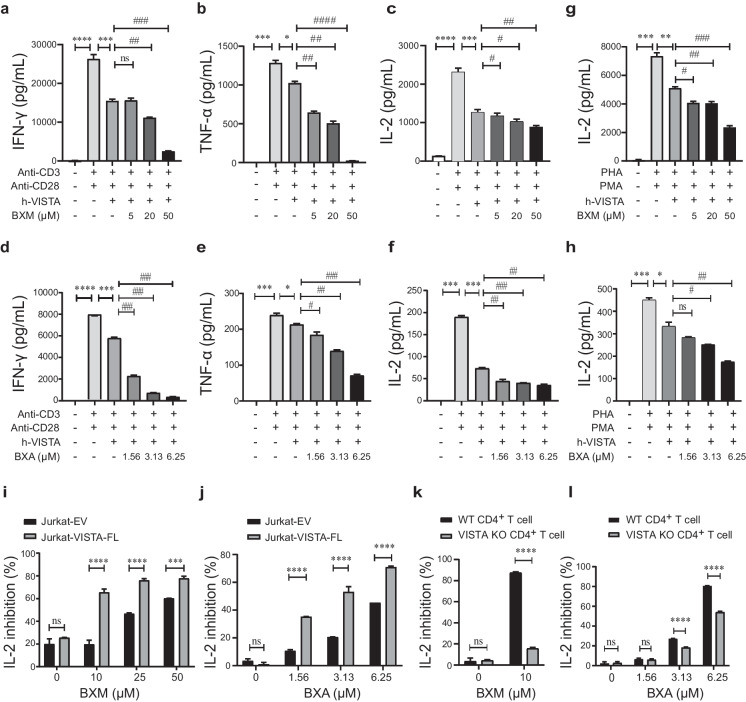
BXM as well as BXA was acting through VISTA in cells. **a**–**f** A total of 1 × 10^5^ PBMCs were incubated with an immobilized anti-human CD3 antibody (2.5 μg·mL^−1^), anti-human CD28 antibody (2.5 μg·mL^−1^), and human VISTA at 2.5 μg·mL^−1^ with or without compound BXM (**a**–**c**) or BXA (**d**–**f**) as indicated. The levels of IFN-γ (**a**, **d**), TNF-α (**b**, **e**), and IL-2 (**c**, **f**) in the cell culture supernatants were measured at 48 h using ELISA kits. The human VISTA protein at 2.5 μg·mL^−1^ was coated on 96-well flat-bottom plates. **g**, **h** The human VISTA protein at 2.5 μg·mL^−1^ was coated on 96-well flat-bottom plates. Jurkat cells (2 × 10^4^ cells/well) were stimulated with PMA (1 ng·mL^−1^) and PHA (6 μg·ml^−1^) with or without BXM or BXA as indicated. The levels of IL-2 in the culture medium (48 h) were analyzed by ELISA. **i**, **j** The levels of IL-2 were analyzed by ELISA in Jurkat cells overexpression of VISTA (Jurkat-VSITA-FL) and control cell (Jurkat-EV) with treatment same to **g** and **h**. **k**, **l** Purified primary murine CD4^+^ T cells (1 × 10^5^ cells/well) from the spleen of WT or VISTA KO mice were cultured in 96-well flat-bottom plates in the presence of anti-CD3 (2.5 μg·mL^−^.^1^). BXM and BXA were added as indicated, and culture supernatants were collected at 72 h. The levels of IL-2 analyzed by ELISA. Data are shown as means ± SD (*n* = 3). ns: not significant, *P < 0.05, **P < 0.01, ***P < 0.001, ****P < 0.0001

To evaluate the biological activities of BXM as well as BXA targeting VISTA, we used the well-established T-cell systems by which the function of VISTA was first identified, and the VISTA modulators were assessed previously by us and others (Hu et al. 2021; Prodeus et al. 2017). Cytokine levels in the PBMCs treated with VISTA and BXM or BXA were determined first. In line with our previously reported, the secretion of IFN-γ, TNF-α, and IL-2 induced by anti-human CD3 and CD28 antibodies was suppressed upon VISTA proteins treatment, which was further reduced after BXM or BXA treatment (Fig. 6a–f). Additionally, both BXM and BXA further inhibited the proliferation of PBMCs when receiving the stimulation of anti-human-CD3 without cytotoxicity (Supplementary Fig. S2a, b, e, f). In Jurkat cells, VISTA is known to inhibit IL-2 production in the presence of PMA and PHA. BXM or BXA treatment further prevented the production of IL-2 (Fig. 6g, h). We also found that BXM or BXA caused a greater inhibition of IL-2 secretion induced by PHA and PMA in Jurkat cells overexpressing VISTA compared to control cells (Fig. 6i, j). These results suggest that BXM and BXA can enhance VISTA function on T cells. To further assess the specificity of BXM and BXA in adjusting VISTA’s actions, we isolated CD4^+^ T cells from the spleens of the VISTA KO and WT mice. IL-2 production was suppressed by activated WT CD4^+^ T cells with BXM treatment. However, this effect was almost lost in the VISTA KO CD4^+^ T cells (Fig. 6k). The suppression of IL-2 secretion caused by BXA treatment was also significantly decreased in the VISTA KO CD4^+^ T cells compared to the WT group (Fig. 6l). These results indicate that BXM as well as BXA was acting through VISTA in cells. Thus, BXM and BXA serve as VISTA agonists.

### BXM alleviates OVA-induced airway inflammation

**Fig. 7:**
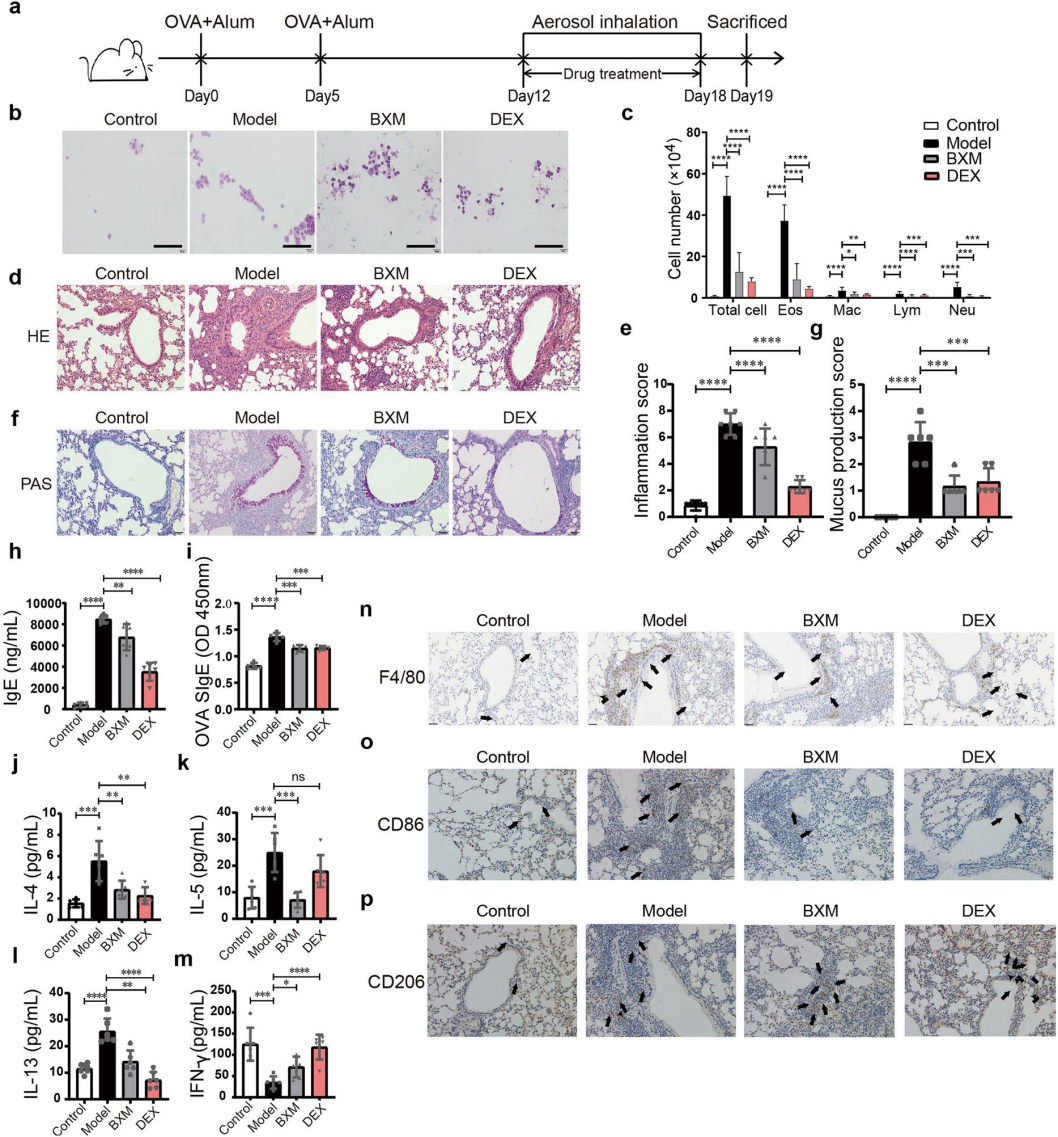
BXM alleviates OVA-induced airway inflammation and macrophage polarization in mice. **a** Experimental protocol for the drug treatment of experimental asthma. **b**, **c** BALF was collected and stained for leukocyte counts. The scale bar represents 80 μm. **d**–**g** Representative H&E staining (**d**) and PAS staining (**f**) of lung tissues. Quantitative analysis of inflammatory (**e**) and PAS^+^ cells (**g**) in lung tissue. **h**–**m** ELISA analysis of total IgE (**h**) and OVA-specific IgE (**i**) levels in serum and IL-4 (**j**), IL-5 (**k**), IL-13 (**l**), and IFN-γ (**m**) in the BALF. Data are shown as means ± SD (*n* = 6). ns: not significant, *P < 0.05, **P < 0.01, ***P < 0.001, ****P < 0.0001. **n**–**p** Representative images of IHC for F4/80 (**n**), CD86 (**o**), and CD206 (**p**) in lung tissue from OVA-induced asthma model. Black arrows showing positive cells. Scale bars are 50 μm

To study the effects of BXM in vivo, mice were treated with BXM during OVA inhalation to induce asthma (Fig. 7a). Similar to the positive control dexamethasone (DEX) treatment, BXM treatment also dramatically decreased total inflammatory cells, eosinophils, macrophages, lymphocytes, and neutrophils in the collected BALF compared with the model group (Fig. 7b, c). Meanwhile, inflammatory cell infiltration around the bronchi and vascular (Fig. 7d, e), overproduction of mucus (Fig. 7f, g), and serum IgE levels and OVA-specific IgE antibody levels were both markedly reduced (Fig. 7h, i). These results suggest that BXM treatment ameliorates OVA-induced lung inflammation.

As expected, we found that BXM administration significantly reduced the OVA-induced generation of cytokines IL-4, IL-5, and IL-13 (Fig. 7j–l). On the contrary, Th1-type cytokine IFN-γ was somewhat restored in the BXM group (Fig. 7m). These results indicate that BXM inhibits Th2-mediated responses in an asthmatic mouse model.

### BXM regulates macrophage polarization in OVA-induced mice

To investigate BXM treatment’s impact on macrophage polarization in an asthma model in more detail. Immunohistochemical staining of F4/80, CD86, and CD206 was used as the indicators of total macrophages and M1 and M2 macrophages respectively. Compared with the model group, we found that BXM treatment obviously decreased the total macrophages and the M1 macrophages and slightly increased the M2 macrophages (Fig. 7n–p). Interestingly, DEX treatment exhibited similar effects (Fig. 7n–p), which might reflect the in vitro studies showing that DEX could shift M1 to M2-like macrophages (Luvanda et al. 2021). Altogether, these findings imply that BXM may inhibit the M1 polarization and promote the M2 polarization during OVA-induced asthma.

### Ameliorative effect of BXM is attenuated in the presence of anti-VISTA antibody

**Fig. 8:**
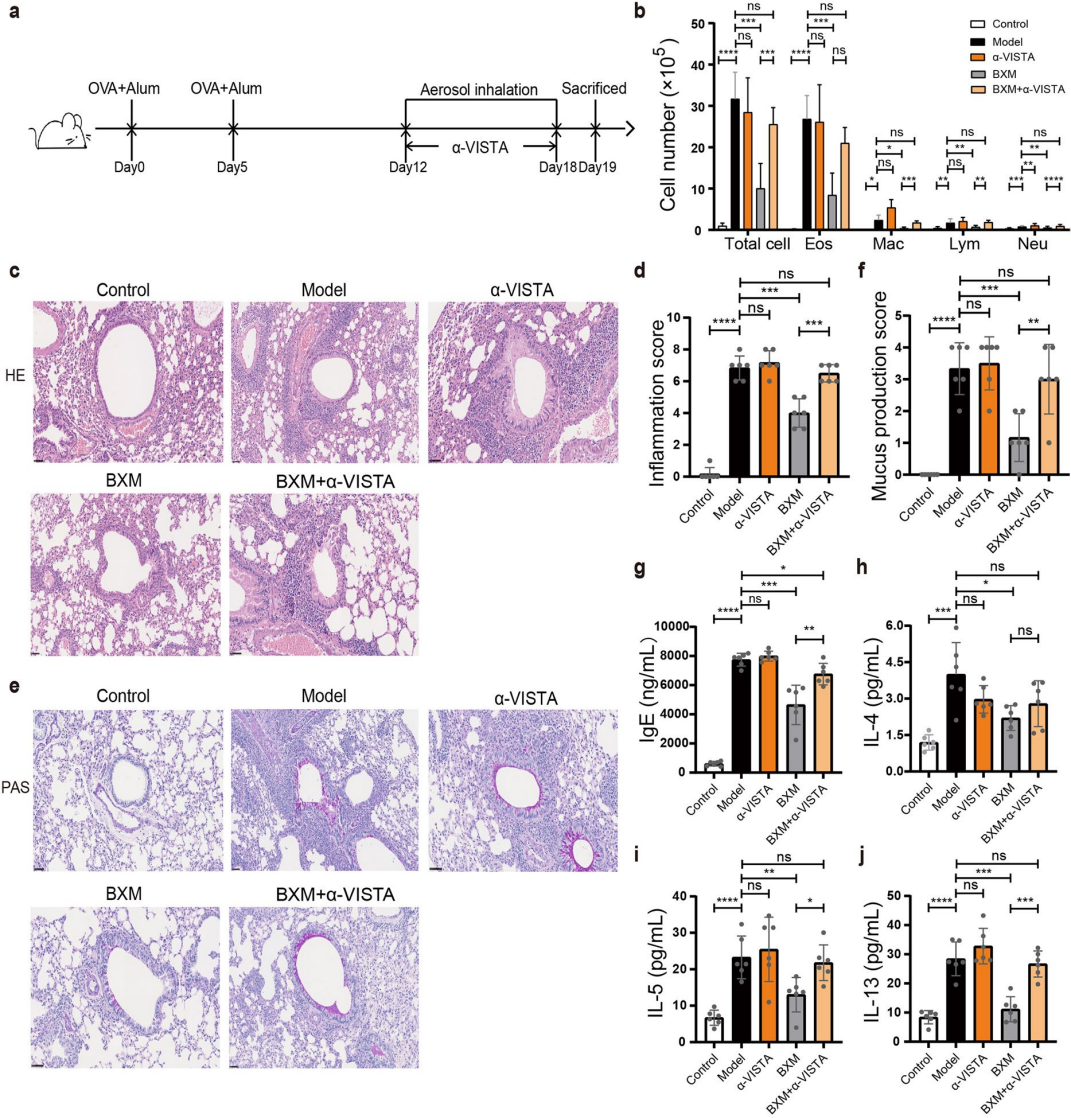
BXM regulates macrophage polarization in OVA-induced mice. **a** Experimental protocol to induce experimental asthma and treatment with anti-VISTA mAb. **b** The total cell numbers and proportions of each leukocyte fraction in the BAL fluids. **c**–**f** Lung tissue histology with H&E staining (**c**) and PAS staining (**e**) and quantitative analysis of inflammation (**d**) and PAS.^+^ cells (**f**). Scale bars are 50 μm. **g**–**j** Total serum IgE (**g**) and levels of IL-4 (**h**), IL-5 (**i**), and IL-13 (**j**) in BALF were examined by ELISA. Data are shown as means ± SD (*n* = 6). ns: not significant, *P < 0.05, **P < 0.01, ***P < 0.001, ****P < 0.0001

To investigate BXM’s protective effects based on VISTA targeting, we treated OVA-induced asthma mice with the anti-VISTA antibody on days 12, 14, 16, and 18 (Fig. 8a). We found that the ameliorative effects of BXM observed a reduction in immune cells in BALF fluid (Fig. 8b), inflammatory cell infiltration, and overproduction of mucus (Fig. 8c–f), and levels of IgE in serum and IL-4, IL-5, and IL-13 in BLAF were notably attenuated when administering VISTA antibodies simultaneously (Fig. 8g–j). Above results indicate that BXM ameliorates asthma symptoms depending on the modulation of VISTA. Interestingly, unlike VISTA deletion, this wildly used anti-VISTA antibody itself did not obviously exacerbate asthma symptoms (Fig. 8b–j). Similar phenomenon was also observed by the Chen group when they used their agonistic VISTA antibodies (Liu et al. 2018).

## Discussion

In this investigation, we discovered that VISTA KO mice exhibited more serious pulmonary inflammation in the OVA-induced allergic asthma mouse model, which is in line with the earlier findings (Liu et al. 2018). To explore which cell types in OVA-induced asthma models are mainly affected by VISTA, we used CyTOF technology to analyze the lung tissues and found macrophages. Our results also showed that macrophages increased in the BALF from VISTA KO mice, which is inconsistent with a previous study reported by the Chen group (Liu et al. 2018). However, Ohno et al. using an anti-VISTA monoclonal antibody (MIH63) also revealed an increase in macrophages in the BALF after VISTA blockade.

In asthma patients, more macrophage numbers were observed in their lungs (Veen et al. 2020). Depletion of macrophage has been reported to reduce inflammatory infiltration and airway hyperresponsiveness induced by treatment with OVA (Hadjigol et al. 2020). As reported, macrophages are a group of highly heterogeneous cells. Upon exposure to the local stimulus, macrophages recruited could polarize into either M1 or M2 (Saradna et al. 2018; Deng et al. 2019). Generally, M1 was considered to play an inflammatory role and M2 to play an immunosuppressive role respectively (Saradna et al. 2018). Here, we found that VISTA deletion promotes the M1 polarization and inhibits the M2 polarization both in BMDMs and in OVA-induced allergic asthma, with reduced expression of inflammatory-related genes, such as Il1b and Tnf-a. Previous studies pointed to the two macrophage states mirroring Th1-Th2 polarization of T cells and increased M2 polarization contributing to the pathology of type 2 (T2) asthma (Melgert et al. 2011; Girodet et al. 2016; Mantovani et al. 2013; Sica and Mantovani 2012). However, in our study, VISTA loss resulted in the upregulation of Th2 cytokines (IL-5 and IL-13), which was not accompanied by an increase in M2 polarization, but rather a decrease in M2 polarization. Above results support that VISTA is involved in controlling pulmonary inflammation in asthma by directly regulating the polarization of macrophages. Nevertheless, our results did not exclude the possibility that VISTA regulates CD4^+^ T-cell differentiation or other cell types and participates in the regulation of lung inflammation. So far as we know, the exact VISTA signaling pathway has not been identified. ElTanbouly et al. reported that an anti-VISTA agonist antibody (8G8) induced macrophage re-programming to augment lipopolysaccharide (LPS) tolerance, which was mediated by a combination of mediators engaged in the development of both macrophage tolerance (IL-10, A20, IRG1, miR221) and transcription factors which lead to an anti-inflammatory profile (e.g., NFKB1, IRF5, IRF8) (ElTanbouly et al. 2020). Studies have reported the overlapping mediators between M2 polarization and LPS tolerance including IL-10, A20, IRG1, miR-221, and MerTK (ElTanbouly et al. 2020; Porta et al. 2009). Thus, whether VISTA regulates macrophage polarization via these mediators in allergic asthma needs future work to verify. Nzeteu et al. reported that M1 and M2 express comparable VISTA on the surface, but M1 releases more soluble VISTA (an extracellular domain) than M2 in vitro (Noubissi Nzeteu et al. 2022). However, the physiological effect of the soluble VISTA is nearly unknown. Interestingly, we also found that a subgroup of Ly6C^+^ macrophages significantly decreased in the lungs of VISTA KO mice. Previous studies have shown a pro-inflammatory role of Ly6C^+^ macrophages in a range of illnesses, such as diabetes (Kimball et al. 2018), liver injury (Cohen et al. 2021), and atherosclerosis (Chung et al. 2015). An increase in Ly6C^+^ inflammatory monocytes in OVA-induced mice was reported previously (Hurdayal et al. 2019). What is the role of Ly6C^+^ macrophages in OVA-induced asthma and how VISTA affects Ly6C^+^ macrophages deserve further study in the future.

We previously reported a new modulator of VISTA, M351-0056 based on our precise virtual VISTA 3D model (Hu et al. 2021). By using the same model, but a FDA-approved small-molecule drug bank, we discovered two drugs as potential small-molecule ligands of VISTA. One is imatinib, which we recently published to be effective in treating systemic lupus erythematosus (SLE) by enhancing VISTA function. In this study, we focused on the other one, BXM, an agent for the treatment of influenza virus infection, as well as its active form BXA. To verify the function and specificity of BXM for VISTA, we performed a series of verifications at the cellular level as before (Hu et al. 2021; Yang et al. 2023) and identified BXM and BXA as VISTA agonists. The original target of BXM is cap-dependent endonuclease (CEN), involved in RNA replication, which is specific for viruses, and no CEN enzyme is encoded in the human genome (Toba et al. 2022). Notably, BXM treatment alleviated OVA-induced lung inflammation in mice, associated with less M1 and more M2 polarization. The ameliorative effect of BXM was attenuated by anti-VISTA antibody treatment, further indicating that BXM worked through enhancing VISTA function. Interestingly, unlike VISTA knockout, the well-used anti-VISTA antibody (13F3) (Srivastava et al. 1950; Rosenbaum et al. 2020; Mercier et al. 2014), being wildly reported to block the VISTA on T cells, itself did not obviously exacerbate inflammatory symptoms. It is possible that the ligand or binding proteins for VISTA on the macrophage surface may be different from those on other cells, such as T cells. Thus, this antibody binding to VISTA may not influence their interaction, while this binding may influence BXM/BXA binding to VISTA. This speculation was supported by a previous study from the Chen group (Liu et al. 2018). As they reported, their previous agonistic VISTA antibodies (MH5A and mam82), functioned well to T cells, but did not improve OVA-induced airway inflammation, so they developed a new one and found that worked well (Liu et al. 2018).

In conclusion, this research revealed that the regulation of macrophage polarization at least is one of the mechanisms by which VISTA participates in OVA-induced asthma progression. Besides, to our knowledge, this is the first report showing that BXM, as an anti-influenza drug, is an agonist of VISTA and might be repositioned as a new treatment for allergic asthma.

## Supplementary Information

Below is the link to the electronic supplementary material.

Supplementary file1 (DOCX 18 KB)

Supplementary file2 (DOCX 262 KB)

## Data Availability

The data that support the findings of this study are available from the corresponding author upon reasonable request. Some data may not be made available because of privacy or ethical restrictions.

## References

[CR1] Adams BK, Cydulka RK. Asthma evaluation and management. Emerg Med Clin North Am. 2003;21(2):315–30. 12793616 10.1016/s0733-8627(03)00015-4

[CR2] Ando Y, Noshi T, Sato K, Ishibashi T, Yoshida Y, Hasegawa T, et al. Pharmacokinetic and pharmacodynamic analysis of baloxavir marboxil, a novel cap-dependent endonuclease inhibitor, in a murine model of influenza virus infection. J Antimicrob Chemother. 2021;76(1):189–98. 33035324 10.1093/jac/dkaa393 PMC7729387

[CR3] Ceeraz S, Eszterhas SK, Sergent PA, Armstrong DA, Ashare A, Broughton T, et al. VISTA deficiency attenuates antibody-induced arthritis and alters macrophage gene expression in response to simulated immune complexes. Arthritis Res Ther. 2017;19(1):270. 10.1186/s13075-017-1474-y PMC5721690 29216931

[CR4] Chung Y, Hong JY, Lei J, Chen Q, Bentley JK, Hershenson MB. Rhinovirus infection induces interleukin-13 production from CD11b-positive, M2-polarized exudative macrophages. Am J Respir Cell Mol Biol. 2015;52(2):205–16. 25029349 10.1165/rcmb.2014-0068OC PMC4370246

[CR5] Cohen K, Mouhadeb O, Ben Shlomo S, Langer M, Neumann A, Erez N, et al. COMMD10 is critical for Kupffer cell survival and controls Ly6C(hi) monocyte differentiation and inflammation in the injured liver. Cell Rep. 2021;37(7):110026. 34788631 10.1016/j.celrep.2021.110026 PMC8998879

[CR6] Cottini M, Lombardi C, Passalacqua G, Bagnasco D, Berti A, Comberiati P, et al. Small airways: the “silent zone” of 2021 GINA report? Front Med (lausanne). 2022;9:884679. 35677830 10.3389/fmed.2022.884679 PMC9168121

[CR7] Deng R, Chen X, Zhang Y, Bian F, Gao N, Hu J, et al. Short ragweed pollen promotes M2 macrophage polarization via TSLP/TSLPR/OX40L signaling in allergic inflammation. Mucosal Immunol. 2019;12(5):1141–9. 31350466 10.1038/s41385-019-0187-8 PMC7285437

[CR8] Deng L, Jian Z, Xu T, Li F, Deng H, Zhou Y, et al. Macrophage polarization: an important candidate regulator for lung diseases. Molecules. 2023;28(5):2379. 10.3390/molecules28052379 PMC10005642 36903624

[CR9] ElTanbouly MA, Schaafsma E, Smits NC, Shah P, Cheng C, Burns C, et al. VISTA Re-programs macrophage biology through the combined regulation of tolerance and anti-inflammatory pathways. Front Immunol. 2020;11:580187. 33178206 10.3389/fimmu.2020.580187 PMC7593571

[CR10] ElTanbouly MA, Zhao Y, Nowak E, Li J, Schaafsma E, Le Mercier I, et al. VISTA is a checkpoint regulator for naive T cell quiescence and peripheral tolerance. Science (New York, NY). 2020;367(6475). 10.1126/science.aay0524 PMC7391053 31949051

[CR11] Fisher CE, Ahmad SA, Fitch PM, Lamb JR, Howie SE. FITC-induced murine pulmonary inflammation: CC10 up-regulation and concurrent Shh expression. Cell Biol Int. 2005;29(10):868–76. 16150617 10.1016/j.cellbi.2005.07.002

[CR12] Flies DB, Higuchi T, Chen L. Mechanistic assessment of PD-1H coinhibitory receptor-induced T cell tolerance to allogeneic antigens. J Immunol (Baltimore, Md: 1950). 2015;194(11):5294–304. 10.4049/jimmunol.1402648 PMC4433880 25917101

[CR13] Gao JJ, Ward JF, Pettaway CA, Shi LZ, Subudhi SK, Vence LM, et al. VISTA is an inhibitory immune checkpoint that is increased after ipilimumab therapy in patients with prostate cancer. Nat Med. 2017;23(5):551. 28346412 10.1038/nm.4308 PMC5466900

[CR14] Girodet PO, Nguyen D, Mancini JD, Hundal M, Zhou X, Israel E, et al. Alternative macrophage activation is increased in asthma. Am J Respir Cell Mol Biol. 2016;55(4):467–75. 27248771 10.1165/rcmb.2015-0295OC PMC5070104

[CR15] Hadjigol S, Netto KG, Maltby S, Tay HL, Nguyen TH, Hansbro NG, et al. Lipopolysaccharide induces steroid-resistant exacerbations in a mouse model of allergic airway disease collectively through IL-13 and pulmonary macrophage activation. Clin Exp Allergy. 2020;50(1):82–94. 31579973 10.1111/cea.13505

[CR16] Halim TY, Krauss RH, Sun AC, Takei F. Lung natural helper cells are a critical source of Th2 cell-type cytokines in protease allergen-induced airway inflammation. Immunity. 2012;36(3):451–63. 22425247 10.1016/j.immuni.2011.12.020

[CR17] Han X, Vesely MD, Yang WD, Sanmamed MF, Badri T, Alawa J, et al. PD-1H (VISTA)-mediated suppression of autoimmunity in systemic and cutaneous lupus erythematosus. Sci Transl Med. 2019;11(522):eaax1159. 10.1126/scitranslmed.aax1159 31826980

[CR18] Haspeslagh E, van Helden MJ, Deswarte K, De Prijck S, van Moorleghem J, Boon L, et al. Role of NKp46(+) natural killer cells in house dust mite-driven asthma. EMBO Mol Med. 2018;10(4):e8657. 10.15252/emmm.201708657 PMC5887908 29444897

[CR19] Hu XL, Qie CX, Jiang JW, Xie XX, Chen WT, Liu WM, et al. M351–0056 is a novel low MW compound modulating the actions of the immune-checkpoint protein VISTA. Brit J Pharmacol. 2021;178(6):1445–58. 33450048 10.1111/bph.15357 PMC9328666

[CR20] Hurdayal R, Nieuwenhuizen NE, Khutlang R, Brombacher F. Inflammatory dendritic cells, regulated by IL-4 receptor alpha signaling, control replication, and dissemination of Leishmania major in mice. Front Cell Infect Microbiol. 2019;9:479. 32039054 10.3389/fcimb.2019.00479 PMC6992597

[CR21] Johnston RJ, Su LJ, Pinckney J, Critton D, Boyer E, Krishnakumar A, et al. VISTA is an acidic pH-selective ligand for PSGL-1. Nature. 2019;574(7779):565–70. 31645726 10.1038/s41586-019-1674-5

[CR22] Kardas G, Panek M, Kuna P, Damianski P, Kupczyk M. Monoclonal antibodies in the management of asthma: dead ends, current status and future perspectives. Front Immunol. 2022;13:983852. 36561741 10.3389/fimmu.2022.983852 PMC9763885

[CR23] Kimball A, Schaller M, Joshi A, Davis FM, denDekker A, Boniakowski A, et al. Ly6C(Hi) blood monocyte/macrophage drive chronic inflammation and impair wound healing in diabetes mellitus. Arterioscler Thromb Vasc Biol. 2018;38(5):1102–14. 29496661 10.1161/ATVBAHA.118.310703 PMC5920725

[CR24] Koski RR, Grzegorczyk KM. Comparison of monoclonal antibodies for treatment of uncontrolled eosinophilic asthma. J Pharm Pract. 2020;33(4):513–22. 31046541 10.1177/0897190019840597

[CR25] Kuo CH, Tsai ML, Li CH, Hsiao HP, Chao MC, Lee MS, et al. Altered pattern of macrophage polarization as a biomarker for severity of childhood asthma. J Inflamm Res. 2021;14:6011–23. 34819741 10.2147/JIR.S319754 PMC8608023

[CR26] Le Mercier I, Chen W, Lines JL, Day M, Li J, Sergent P, et al. VISTA regulates the development of protective antitumor immunity. Can Res. 2014;74(7):1933–44. 10.1158/0008-5472.CAN-13-1506 PMC4116689 24691994

[CR27] Li L, Wei C, Cai S, Fang L. TRPM7 modulates macrophage polarization by STAT1/STAT6 pathways in RAW264.7 cells. Biochem Biophys Res Commun. 2020;533(4):692–7. 33153718 10.1016/j.bbrc.2020.10.062

[CR28] Li X, Yang S, Zhang H, Liu X, Gao Y, Chen Y, et al. Discovery of orally bioavailable N-benzylpiperidinol derivatives as potent and selective USP7 inhibitors with in vivo antitumor immunity activity against colon cancer. J Med Chem. 2022;65(24):16622–39. 36454192 10.1021/acs.jmedchem.2c01444

[CR29] Liu Y, Kloc M, Li XC. Macrophages as effectors of acute and chronic allograft injury. Curr Transplant Rep. 2016;3(4):303–12. 28546901 10.1007/s40472-016-0130-9 PMC5440082

[CR30] Liu HF, Li X, Hu L, Zhu M, He BL, Luo LQ, et al. A crucial role of the PD-1H coinhibitory receptor in suppressing experimental asthma. Cell Mol Immunol. 2018;15(9):838–45. 28479600 10.1038/cmi.2017.16 PMC6203798

[CR31] Luvanda MK, Posch W, Vosper J, Zaderer V, Noureen A, Lass-Florl C, et al. Dexamethasone promotes Aspergillus fumigatus growth in macrophages by triggering M2 repolarization via targeting PKM2. J Fungi (Basel). 2021;7(2):70. 10.3390/jof7020070 PMC7909285 33498318

[CR32] Mantovani A, Biswas SK, Galdiero MR, Sica A, Locati M. Macrophage plasticity and polarization in tissue repair and remodelling. J Pathol. 2013;229(2):176–85. 23096265 10.1002/path.4133

[CR33] Melgert BN, ten Hacken NH, Rutgers B, Timens W, Postma DS, Hylkema MN. More alternative activation of macrophages in lungs of asthmatic patients. J Allergy Clin Immunol. 2011;127(3):831–3. 21167569 10.1016/j.jaci.2010.10.045

[CR34] Miki H, Pei H, Gracias DT, Linden J, Croft M. Clearance of apoptotic cells by lung alveolar macrophages prevents development of house dust mite-induced asthmatic lung inflammation. J Allergy Clin Immunol. 2021;147(3):1087-92.e3. 33065121 10.1016/j.jaci.2020.10.005 PMC7940554

[CR35] Noubissi Nzeteu GA, Schlichtner S, David S, Ruppenstein A, Fasler-Kan E, Raap U, et al. Macrophage differentiation and polarization regulate the release of the immune checkpoint protein V-domain Ig suppressor of T cell activation. Front Immunol. 2022;13:837097. 35634346 10.3389/fimmu.2022.837097 PMC9132587

[CR36] Ohno T, Zhang C, Kondo Y, Kang S, Furusawa E, Tsuchiya K, et al. The immune checkpoint molecule VISTA regulates allergen-specific Th2-mediated immune responses. Int Immunol. 2018;30(1):3–11. 29267882 10.1093/intimm/dxx070

[CR37] Pedersen SE, Hurd SS, Lemanske RF Jr, Becker A, Zar HJ, Sly PD, et al. Global strategy for the diagnosis and management of asthma in children 5 years and younger. Pediatr Pulmonol. 2011;46(1):1–17. 20963782 10.1002/ppul.21321

[CR38] Porta C, Rimoldi M, Raes G, Brys L, Ghezzi P, Di Liberto D, et al. Tolerance and M2 (alternative) macrophage polarization are related processes orchestrated by p50 nuclear factor kappaB. Proc Natl Acad Sci U S A. 2009;106(35):14978–83. 19706447 10.1073/pnas.0809784106 PMC2736429

[CR39] Prodeus A, Abdul-Wahid A, Sparkes A, Fischer NW, Cydzik M, Chiang N, et al. VISTA.COMP - an engineered checkpoint receptor agonist that potently suppresses T cell-mediated immune responses. JCI Insight. 2017;2(18):e94308. 10.1172/jci.insight.94308 PMC5621893 28931757

[CR40] Rigamonti E, Zordan P, Sciorati C, Rovere-Querini P, Brunelli S. Macrophage plasticity in skeletal muscle repair. Biomed Res Int. 2014;2014:560629. 24860823 10.1155/2014/560629 PMC4016840

[CR41] Rosenbaum SR, Knecht M, Mollaee M, Zhong Z, Erkes DA, McCue PA, et al. FOXD3 regulates VISTA expression in melanoma. Cell Rep. 2020;30(2):510-24.e6. 31940493 10.1016/j.celrep.2019.12.036 PMC6995351

[CR42] Saradna A, Do DC, Kumar S, Fu QL, Gao P. Macrophage polarization and allergic asthma. Transl Res. 2018;191:1–14. 29066321 10.1016/j.trsl.2017.09.002 PMC5776696

[CR43] Sica A, Mantovani A. Macrophage plasticity and polarization: in vivo veritas. J Clin Invest. 2012;122(3):787–95. 22378047 10.1172/JCI59643 PMC3287223

[CR44] Song YD, Li XZ, Wu YX, Shen Y, Liu FF, Gao PP, et al. Emodin alleviates alternatively activated macrophage and asthmatic airway inflammation in a murine asthma model. Acta Pharmacol Sin. 2018;39(8):1317–25. 29417945 10.1038/aps.2017.147 PMC6289379

[CR45] Srivastava R, Hernandez-Ruiz M, Khan AA, Fouladi MA, Kim GJ, Ly VT, et al. CXCL17 chemokine-dependent mobilization of CXCR8(+)CD8(+) effector memory and tissue-resident memory T cells in the vaginal mucosa is associated with protection against genital herpes. J Immunol (Baltimore, Md: 1950). 2018;200(8):2915–26. 10.4049/jimmunol.1701474 PMC5893430 29549178

[CR46] Toba S, Sato A, Kawai M, Taoda Y, Unoh Y, Kusakabe S, et al. Identification of cap-dependent endonuclease inhibitors with broad-spectrum activity against bunyaviruses. Proc Natl Acad Sci U S A. 2022;119(36):e2206104119. 36037386 10.1073/pnas.2206104119 PMC9457168

[CR47] van der Veen TA, de Groot LES, Melgert BN. The different faces of the macrophage in asthma. Curr Opin Pulm Med. 2020;26(1):62–8. 31703000 10.1097/MCP.0000000000000647 PMC6903353

[CR48] Wang L, Le Mercier I, Putra J, Chen W, Liu J, Schenk AD, et al. Disruption of the immune-checkpoint VISTA gene imparts a proinflammatory phenotype with predisposition to the development of autoimmunity. Proc Natl Acad Sci U S A. 2014;111(41):14846–51. 25267631 10.1073/pnas.1407447111 PMC4205642

[CR49] Wang J, Wu G, Manick B, Hernandez V, Renelt M, Erickson C, et al. VSIG-3 as a ligand of VISTA inhibits human T-cell function. Immunology. 2019;156(1):74–85. 30220083 10.1111/imm.13001 PMC6283650

[CR50] Xie X, Chen C, Chen W, Jiang J, Wang L, Li T, et al. Structural basis of VSIG3: the ligand for VISTA. Front Immunol. 2021;12:625808. 33841409 10.3389/fimmu.2021.625808 PMC8027081

[CR51] Xu WW, Dong J, Zheng YW, Zhou J, Yuan Y, Ta HM, et al. Immune-checkpoint protein VISTA regulates antitumor immunity by controlling myeloid cell-mediated inflammation and immunosuppression. Cancer Immunol Res. 2019;7(9):1497–510. 31340983 10.1158/2326-6066.CIR-18-0489 PMC6726548

[CR52] Yang T. Baloxavir marboxil: the first cap-dependent endonuclease inhibitor for the treatment of influenza. Ann Pharmacother. 2019;53(7):754–9. 30674196 10.1177/1060028019826565

[CR53] Yang L, Zhang T, Wang P, Chen W, Liu W, He X, et al. Imatinib and M351–0056 enhance the function of VISTA and ameliorate the development of SLE via IFN-I and noncanonical NF-κB pathway. Cell Biol Toxicol. 2023;39(6):3287–304. 10.1007/s10565-023-09833-6 37804401

[CR54] Yuan L, Tatineni J, Mahoney KM, Freeman GJ. VISTA: a mediator of quiescence and a promising target in cancer immunotherapy. Trends Immunol. 2021;42(3):209–27. 33495077 10.1016/j.it.2020.12.008 PMC8088836

[CR55] Zhang YYF, Xiao YB. Development of VISTA in tumor immunotherapy. J China Pharm Univ. 2022;53(4):400–9 (**Chinese**).

