# Supplementary material for: Repositioning baloxavir marboxil as VISTA agonist that ameliorates experimental asthma
Source: Cell Biol Toxicol. 2024 Feb 10;40(1):12. doi: 10.1007/s10565-024-09852-x (PMC10858940; doi:10.1007/s10565-024-09852-x)
Supplement: Supplementary file 1 — Supplementary file1 (DOCX 18 KB) [file 10565_2024_9852_MOESM1_ESM.docx]

**Supplementary Table S1 Mass cytometry antibody panels**

| serial number | Label | Target | serial number | Label | Target |
| --- | --- | --- | --- | --- | --- |
| 1 | 209Bi | MHCII | 22 | 175Lu | CD127 |
| 2 | 111Cd | CD4 | 23 | 142Nd | CD11c |
| 3 | 112Cd | Ly6C | 24 | 143Nd | CD69 |
| 4 | 113Cd | CD138 | 25 | 144Nd | CD115 |
| 5 | 114Cd | CD44 | 26 | 145Nd | TCRgd |
| 6 | 116Cd | B220 | 27 | 146Nd | CD43 |
| 7 | 161Dy | Tbet | 28 | 148Nd | MerTK |
| 8 | 162Dy | CD103 | 29 | 150Nd | CD27 |
| 9 | 163Dy | BCL6 | 30 | 141Pr | Ly6G |
| 10 | 164Dy | CX3CR1 | 31 | 174Yb | CD5 |
| 11 | 166Er | CD19 | 32 | 176Yb | FceR1 |
| 12 | 167Er | Gata3 | 33 | 147Sm | F4/80 |
| 13 | 168Er | CD8 | 34 | 149Sm | CD25 |
| 14 | 170Er | Siglec1 | 35 | 152Sm | CD3 |
| 15 | 151Eu | CD64 | 36 | 154Sm | CTLA4 |
| 16 | 153Eu | NKp46 | 37 | 159Tb | RORgt |
| 17 | 155Gd | CCR2 | 38 | 169Tm | Ki67 |
| 18 | 156Gd | KLRG1 | 39 | 89Y | CD45 |
| 19 | 158Gd | CD49b | 40 | 171Yb | CD38 |
| 20 | 160Gd | CD62L | 41 | 172Yb | CD11b |
| 21 | 165Ho | Foxp3 | 42 | 173Yb | CD24 |

**Supplementary Table S2 The primer sequences used in this study**

|  | Forward Primer(5’-3’) | Reverse Primer(5’-3’) |
| --- | --- | --- |
| iNOS | GTTCTCAGCCCAACAATACAAGA | GTGGACGGGTCGATGTCAC |
| Arg1 | CTCCAAGCCAAAGTCCTTAGAG | AGGAGCTGTCATTAGGGACATC |
| IL-1β | CAGGCAGGCAGTATCACTCATTG | CGTCACACACCAGCAGGTTATC |
| GAPDH | AGGTCGGTGTGAACGGATTTG | GGGGTCGTTGATGGCAACA |
| CD206 | CTCTGTTCAGCTATTGGACGC | CGGAATTTCTGGGATTCAGCTTC |
| Ym-1 | CAGGTCTGGCAATTCTTCTGAA | GTCTTGCTCATGTGTGTAAGTGA |
| Fizz-1 | CCAATCCAGCTAACTATCCCTCC | ACCCAGTAGCAGTCATCCCA |
