# Supplementary material for: Repositioning baloxavir marboxil as VISTA agonist that ameliorates experimental asthma
Source: Cell Biol Toxicol. 2024 Feb 10;40(1):12. doi: 10.1007/s10565-024-09852-x (PMC10858940; doi:10.1007/s10565-024-09852-x)
Supplement: Supplementary file 2 — Supplementary file2 (DOCX 262 KB) [file 10565_2024_9852_MOESM2_ESM.docx]

**
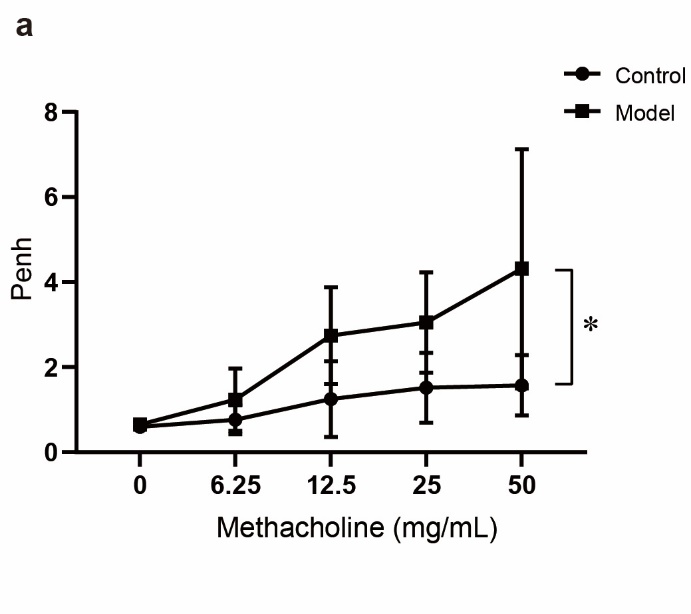
**

**Supplemental fig. 1 OVA inhalation increased pulmonary methacholine hypersensitivity as evdenced by Penh vale**. The noninvasive whole body plethysmography was used to detect Penh within 24 h after the last challenge to reflect AHR. Before testing, the chamber was sealed well and corrected. Then, after adjusting the baseline with phosphate buffered saline, the changes in Penh after acetylcholine chloride (0, 6.25, 12.5, 25, and 50 mg·mL^-1^) atomization inhalation in mice were measured (n=4). *P < 0.05.

**
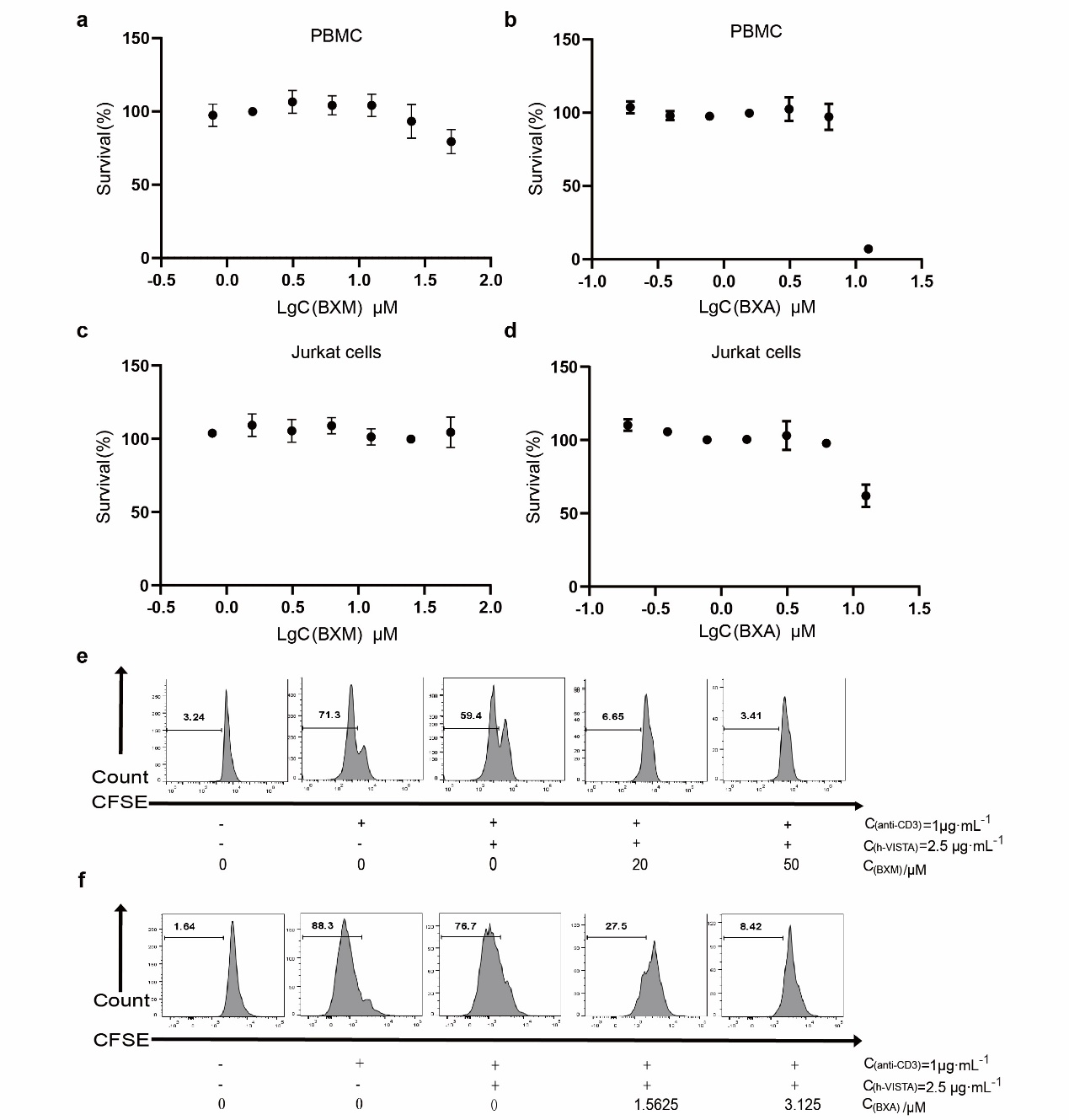
**

**Supplemental fig.2 BXM and BXA further enhanced the inhibition effect of protein human VISTA-ECD on human PBMCs proliferation.** **a**, **b** Detect the cytotoxicity of BXM and BXA on PBMC by CCK8. **c**, **d** Detect the cytotoxicity of BXM and BXA on Jurkat cells by CCK8. **e**, **f** CFSE-labeled PBMCs were stimulated by plate-bound anti-human-CD3 (1 μg·mL^−1^) together with co-absorbed human VISTA-ECD protein at the 2.5 μg·mL^−1^, compound BXM and BXA were added. The proliferation levels of PBMCs were measured on day 5 for CFSE profiles. Data are shown as means ± SD (n=3).
